# Molecular Typing of *Trypanosoma cruzi* Isolates, United States

**DOI:** 10.3201/eid1407.080175

**Published:** 2008-07

**Authors:** Dawn M. Roellig, Emily L. Brown, Christian Barnabé, Michel Tibayrenc, Frank J. Steurer, Michael J. Yabsley

**Affiliations:** * University of Georgia, Athens, Georgia, USA; † Institut de Recherche pour le Developpement, Montpellier, France; ‡ Centers for Disease Control and Prevention, Atlanta, Georgia, USA

**Keywords:** Chagas disease, disease reservoir, genotype, molecular epidemiology, Trypanosoma cruzi, United States, dispatch

## Abstract

Studies have characterized *Trypanosoma cruzi* from parasite-endemic regions. With new human cases, increasing numbers of veterinary cases, and influx of potentially infected immigrants, understanding the ecology of this organism in the United States is imperative. We used a classic typing scheme to determine the lineage of 107 isolates from various hosts.

In Latin America, an estimated 10–12 million persons are infected with *Trypanosoma cruzi*, the etiologic agent of Chagas disease and a major contributor to heart disease within the region. Autochthonous human infections in the United States have been reported in 6 persons, with the most recent case reported from Louisiana (*1*). In addition, the parasite is euryxenous; it is able to infect a broad range of hosts, including domestic dogs, woodrats, raccoons, opossums, armadillos, and nonhuman primates.

Associations between host species and parasite genotype have been suggested and are important in understanding the domestic and sylvatic cycles of *T*. *cruzi* (*2*–*4*). Although studies conducted on US isolates suggest an association between *T*. *cruzi* genotype and host, these studies were limited because of low sample numbers, low host diversity, and narrow geographic distribution (*2*,*4*–*7*). In the current investigation, we used the molecular typing scheme proposed by Brisse et al. (*8*), in which isolates are delineated into 1 of the 6 lineages (types I and IIa–IIe) on the basis of size polymorphisms of several PCR markers. We then expanded characterization of US isolates and show additional evidence for correlations between host specificity and genotype of *T*. *cruzi*.

## The Study

We analyzed 107 isolates of *T*. *cruzi* from multiple species of free-ranging and captive wildlife, domestic animals, triatomine bug vectors, and humans who were autochthonously infected in the United States. Some isolates were obtained as liquid nitrogen–stored parasites from the Centers for Disease Control and Prevention (Atlanta, GA, USA), the Institut Pasteur (Paris, France), and the Southeastern Cooperative Wildlife Disease Study (Athens, GA, USA) and were established in axenic liver infusion tryptose medium as described (*9*). Additional isolates were obtained from wild-trapped animals in axenic liver infusion tryptose medium or canine macrophage cell culture as described (*10*). Isolated DNA was used as template for PCR amplification of 3 gene targets, mini-exon, D7 divergent domain of 24S α rRNA, and 18S rRNA, according to published methods (*8*). Locality data and results of molecular typing of each isolate are shown in the Appendix Table. All animals used in this study were cared for in accordance with the guidelines of the Institutional Animal Care and Use Committee and under animal use protocol approved by the Institutional Animal Care and Use Committee at the University of Georgia.

Only 2 genotypes, *T*. *cruzi* I and *T*. *cruzi* IIa, were detected. Typical amplicon sizes of *T*. *cruzi* I and *T*. *cruzi* IIa isolates from the United States are shown in the Table. Atypical banding patterns and isolates that differ from the standard genotype from a particular host are also represented. With the exception of human isolates, 1 primate isolate, and a few raccoon isolates, placental mammalian isolates, including those from raccoons, domestic dogs, ring-tailed lemurs, and skunks, were characterized as type IIa (Appendix Table). All remaining isolates, including those from Virginia opossums (*Didelphis virginiana*), triatomine vectors, humans, and rhesus macaques from the United States, were identified as type I (Appendix Table).

**Table:** Approximate amplicon sizes of gene targets and lineage determination in *Trypanosoma cruzi*

Strain	Mini-exon, bp	24S α rRNA, bp	18S rRNA, bp	Lineage
FL Opo 15*	350	110	175	I
GA Rac 103*	None	120	155	IIa
FL Rac 5*	400	120	155	IIa
93053103R cl3	350	110	175	I
FL Rac 13	350	110, 120	155, 175	I/IIa†
FL Rac 46	400	110, 120	155	I/IIa†
Griffin Dog	350	110, 120	155	I/IIa†
Monk RH89–40	None	110	155	I/IIa†

*Denotes isolates used as representative banding patterns seen for classic lineage typing. †Because of atypical banding patterns, a clear definition of an isolate as type I vs. type IIa could not be obtained.

## Conclusions

In contrast to studies conducted on South American isolates, for which 6 genotypes of *T*. *cruzi* have been identified, only 2 genotypes (I and IIa) were identified in the current study. These data support results of investigations in Central America and Mexico in which a paucity of genotypes was found (*11*,*12*). Many investigations on *T*. *cruzi* evolutionary ecology have shown strict host–parasite specificity in regard to host species and parasite genotype (*2*–*4*), although exceptions have been observed. The presence of only 2 genotypes in the United States could be caused by a lack of introduction of other genotypes or a lower diversity of natural reservoir hosts for *T*. *cruzi* than in South America. A recent analysis of *T*. *cruzi* hosts in North and South America indicated that >48 host species representing 17 families were infected with >1 of the 6 strains (*4*). Only 6 of these hosts have established populations in the United States, and US isolates from these species were only characterized as types I or IIa (*4*).

Our data for US isolates correspond with those of previous studies in which *Didelphis* spp. are reservoirs for type I *T*. *cruzi* (*4*); no infections with type II parasites were observed. The Virginia opossum (and its ancestors), which is the only marsupial present in the United States (it migrated from South America ≈4.5 million years ago), is a possible host for *T*. *cruzi* I. This evidence suggests that *T*. *cruzi* was not recently introduced into North America or the United States (*5*). Additionally, sufficient time may have passed for random and rare genetic exchange events to occur independent of those found in South American isolates (*13*), enabling the lineage to infect atypical reservoirs (i.e., raccoons) in North America.

The second major natural reservoir of *T*. *cruzi* in the United States is the raccoon. In general, the nonprimate placental mammals in our study were infected with type IIa, a strain that is commonly found in sylvatic cycles in the Southern Cone of South America. Our data confirm previous typing of US isolates by multilocus enzyme electrophoresis or random amplified polymorphic DNA analysis (*5*), in which 11 raccoons from Georgia were characterized as zymodeme 3 (equivalent to IIa). Although raccoons are predominately infected with *T*. *cruzi* IIa, 4 known exceptions include 3 isolates from Georgia and Florida in the current study and 1 raccoon from Louisiana from a previous study (*5*).These data are in contrast to typing data for Virginia opossum isolates, which have all found *T*. *cruzi* I. This finding suggests that opossums primarily maintain persistent infections with *T*. *cruzi* I.

All characterized human isolates from autochthonous US cases of infection with *T*. *cruzi* are *T*. *cruzi* I. This genotype is predominantly responsible for Chagas disease north of the Amazon Basin and is part of the domiciliary cycle of the parasite. Our findings correspond with data from Mexico where *T*. *cruzi* I is the predominate strain detected in humans (*11*). It would be useful to differentiate biologic characteristics and polymorphisms by using additional gene targets in human type I isolates and compare them with those in opossum, triatomine vectors, and rhesus macaque isolates from the United States. Additionally, comparing these US isolates and Mexican reference strains with those from South America may indicate why type I typically infects humans in North America and multiple strains are found in humans in South America.

Our results provide additional evidence that *T*. *cruzi* has distinct genotypes that preferentially infect 1 host species or a group of hosts. Humans and marsupials are typically infected with type I *T*. *cruzi*, but raccoons, skunks, domestic dogs, and prosimians are typically infected with type IIa. Although we only detected *T*. *cruzi* I in triatomid bugs, other studies have detected *T*. *cruzi* IIa in triatomids from the United States (*5*). The mechanism is unknown by which persistent infections with a particular genotype of *T*. *cruzi* develop in certain hosts. Further analysis of isolates from an increased host diversity and geographic range should be pursued. Determining basic infection dynamics of reservoir hosts experimentally infected with various *T*. *cruzi* genotypes may provide additional insight into the host–parasite dichotomy.

## Supplementary Material

Appendix Table Origin and lineage identification of 107 US isolates of Trypanosoma cruzi used in the study*

## References

[1] Dorn PL, Perniciaro L, Yabsley MJ, Roellig DM, Balsamo G, Diaz J, Autochthonous transmission of *Trypanosoma cruzi*, Louisiana. Emerg Infect Dis. 2007;13:605–7. 17553277 10.3201/eid1304.061002 PMC2725963

[2] Clark CG, Pung OJ. Host specificity of ribosomal DNA variation in sylvatic *Trypanosoma cruzi* from North America. Mol Biochem Parasitol. 1994;66:175–9. 10.1016/0166-6851(94)90045-0 7984184

[3] Briones MR, Souto RP, Stolf BS, Zingales B. The evolution of two *Trypanosoma cruzi* subgroups inferred from rRNA genes can be correlated with the interchange of American mammalian faunas in the Cenozoic and has implications to pathogenicity and host specificity. Mol Biochem Parasitol. 1999;104:219–32. 10.1016/S0166-6851(99)00155-3 10593177

[4] Yeo M, Acost N, Llewellyn M, Sánchez H, Adamson S, Miles GA, Origins of Chagas disease: *Didelphis* species are natural hosts of *Trypanosoma cruzi* I and armadillos hosts of *Trypanosoma cruzi* II, including hybrids. Int J Parasitol. 2005;35:225–33. 10.1016/j.ijpara.2004.10.024 15710443

[5] Barnabé C, Yaeger R, Pung O, Tibayrenc M. *Trypanosoma cruzi*: a considerable phylogenetic divergence indicates that the agent of Chagas disease is indigenous to the native fauna of the United States. Exp Parasitol. 2001;99:73–9. 10.1006/expr.2001.4651 11748960

[6] Miles MA, Souza A, Povoa M, Shaw JJ, Lainson E, Toye PJ. Isozymic heterogeneity of *Trypanosoma cruzi* in the first autochthonous patients with Chagas’ disease in Amazonian Brazil. Nature. 1978;272:819–21. 10.1038/272819a0 417267

[7] Yabsley MJ, Noblet GP. Biological and molecular characterization of a raccoon isolate of *Trypanosoma cruzi* from South Carolina. J Parasitol. 2002;88:1273–6. 12537130 10.1645/0022-3395(2002)088[1273:BAMCOA]2.0.CO;2

[8] Brisse S, Verhoef J, Tibayrenc M. Characterisation of large and small subunit rRNA and mini-exon genes further support the distinction of six *Trypanosoma cruzi* lineages. Int J Parasitol. 2001;31:1218–26. 10.1016/S0020-7519(01)00238-7 11513891

[9] Castellani O, Ribeiro LV, Fernandes JF. Differentiation of *Trypanosoma cruzi* in culture. J Protozool. 1967;14:447–51. 6050651 10.1111/j.1550-7408.1967.tb02024.x

[10] Yabsley MJ, Norton TM, Powell MR, Davidson WR. Molecular and serologic evidence of tick-borne ehrlichiae in three species of lemurs from St. Catherine’s Island, Georgia, USA. J Zoo Wildl Med. 2004;35:503–9. 15732591 10.1638/03-116

[11] Bosseno M-F, Bernabé C, Gastélum EM, Kasten FL, Ramsey J, Espinoza B, Predominance of *Trypanosoma cruzi* lineage I in Mexico. J Clin Microbiol. 2002;40:627–32. 10.1128/JCM.40.2.627-632.2002 11825982 PMC153397

[12] Iwagami M, Higo H, Miura S, Yanagi T, Tada I, Kano S, Molecular phylogeny of *Trypanosoma cruzi* from Central America (Guatemala) and a comparison with South American strains. Parasitol Res. 2007;102:129–34. 10.1007/s00436-007-0739-9 17828552

[13] Machado CA, Ayala FJ. Nucleotide sequences provide evidence of genetic exchange among distantly related lineages of *Trypanosoma cruzi.* Proc Natl Acad Sci U S A. 2001;98:7396–401. 10.1073/pnas.121187198 11416213 PMC34680

[14] de Freitas JM, Augusto-Pinto L, Pimenta JR, Bastos-Rodrigues L, Goncalves VF, Teixeira SM, Ancestral genomes, sex and the population structure of *Trypanosoma cruzi.* PLoS Pathog. 2006;2:e24. 10.1371/journal.ppat.0020024 16609729 PMC1434789

[15] Brisse S, Barnabé C, Tibayrenc M. *Trypanosoma cruzi* clonal diversity: identification of discrete phylogenetic lineages by random amplified polymorphic DNA and multilocus enzyme electrophoresis analysis. Int J Parasitol. 2000;30:35–44. 10.1016/S0020-7519(99)00168-X 10675742

