# Supplementary material for: Molecular Typing of Trypanosoma cruzi Isolates, United States
Source: Emerg Infect Dis. 2008 Jul;14(7):1123–5. doi: 10.3201/eid1407.080175 (PMC2600345; doi:10.3201/eid1407.080175)
Supplement: Appendix Table — Origin and lineage identification of 107 US isolates of Trypanosoma cruzi used in the study* [file 08-0175_appT-s1.pdf]

Appendix Table. Origin and lineage identification of 107 US isolates of *Trypanosoma cruzi* used in the study*

Host	Isolate	Site of origin	Lineage
Human	CA R	California	I
	Corpus Christi†	Corpus Christi, TX	I
	LC *T cruzi*	New Orleans, LA	I
	TC California†	Lake Don Pedro, CA	I
	TX D	Alano, TX	I
Domestic dog	Caesar Dog	Not known	IIa
	Dog Theis†‡§	Not known	IIa
	Griffin Dog	Hillsboro, TN	I/IIa
	OK Dog	Bartlesville, OK	IIa
	Samantha Dog	South Carolina	IIa
	Smokey	South Carolina	IIa
	USA Dog Y†	California	IIa
Virginia opossum	92101601P†	Statesboro, GA	I
	93041401P cl1†	Statesboro, GA	I
	93070103P cl2†	Fort Stewart, GA	I
	FL Opo 15	Maclay State Park, FL	I
	FL Opo 17	Wakulla Springs, FL	I
	FL Opo 18	Wakulla Springs, FL	I
	FL Opo 2	Wakulla Springs, FL	I
	FL Opo 3	Wakulla Springs, FL	I
	FL Opo 717	Tampa, FL	I
	GA Opo 43	Chatham County, GA	I
	GA Opo 75	White Hall, GA	I
	Opossum 1970†	New Orleans, LA	I
	USA Opossum†	Southern Louisiana	I
	AU8	Auburn, AL	I
	FH4	Southern Georgia	I
Raccoon	92122102R†	Statesboro, GA	IIa
	93040701R cl1†	Statesboro, GA	IIa
	93053102R cl4†	Harrold Preserve, GA	IIa
	93053103R cl3	Harrold Preserve, GA	I
	93071502R cl2†	Fort Stewart, GA	IIa
	93072805R cl3†	Fort Stewart, GA	IIa
	FL Rac 13	Maclay State Park, FL	I/IIa
	FL Rac 14	Wakulla Springs, FL	IIa
	FL Rac 15	Wakulla Springs, FL	IIa
	FL Rac 26	Wakulla Springs, FL	IIa
	FL Rac 30	Wakulla Springs, FL	IIa
	FL Rac 38	Maclay State Park, FL	IIa
	FL Rac 4 PAD	Tallahassee, FL	IIa
	FL Rac 40	Wakulla Springs, FL	IIa
	FL Rac 42	Wakulla Springs, FL	IIa
	FL Rac 46	Tall Timbers, FL	IIa
	FL Rac 48	Maclay State Park, FL	IIa
	FL Rac 5	Torreya State Park, FL	IIa
	FL Rac 50	Wakulla Springs, FL	IIa
	FL Rac 51	Wakulla Springs, FL	IIa
	FL Rac 7	Lake Talquin, FL	IIa
	FL Rac 9	Torreya State Park, FL	IIa
	FR36#	Pickens County, SC	IIa
	GA Rac 103	Ossabaw Island, GA	IIa
	GA Rac 104	Ossabaw Island, GA	IIa
	GA Rac 107	Ossabaw Island, GA	IIa
	GA Rac 108	Ossabaw Island, GA	IIa
	GA Rac 111	Ossabaw Island, GA	IIa
	GA Rac 121	Ossabaw Island, GA	IIa
	GA Rac 124	Ossabaw Island, GA	IIa
	GA Rac 134	Whitehall Forest, GA	IIa
	GA Rac 135	Whitehall Forest, GA	IIa
	GA Rac 137	Whitehall Forest, GA	IIa
	GA Rac 141	Whitehall Forest, GA	IIa
	GA Rac 142	Whitehall Forest, GA	IIa
	GA Rac 143	Athens, GA	IIa
	GA Rac 144	Athens, GA	IIa
	GA Rac 147	Woodbine, GA	IIa
	GA Rac 148	Woodbine, GA	IIa
	GA Rac 186	White Hall, GA	IIa

	GA Rac 2	Ludiwici, GA	I
	GA Rac 206	Athens, GA	IIa
	GA Rac 208	White Hall, GA	IIa
	GA Rac 22	Victoria Bryant State Park, GA	IIa
	GA Rac 3	Athens, GA	IIa
	GA Rac 45	Skidaway Island, GA	IIa
	GA Rac 46	Skidaway Island, GA	IIa
	GA Rac 51	Skidaway Island, GA	IIa
	GA Rac 52	Skidaway Island, GA	IIa
	GA Rac 55	Skidaway Island, GA	IIa
	GA Rac 57	Skidaway Island, GA	IIa
	GA Rac 61	Skidaway Island, GA	IIa
	GA Rac 67	Athens, GA	IIa
	GA Rac 68	Athens, GA	IIa
	GA Rac 69	Athens, GA	IIa
	Maryland Rac	Laurel, MD	IIa
	STC 10R cl3†	St. Catherine's Island, GA	IIa
	STC 16R cl1†	St. Catherine's Island, GA	IIa
	STC 33R	St. Catherine's Island, GA	IIa
	STC 35R	St. Catherine's Island, GA	IIa
	STC 39R	St. Catherine's Island, GA	IIa
	STC 54R	St. Catherine's Island, GA	IIa
	STC 9R cl4†	St. Catherine's Island, GA	IIa
	TN Rac 18	Rutherford County, TN	IIa
Triatoma sanguisuga	Florida†	Gainesville, FL	I
	Florida C16¶	Gainesville, FL	I
	Florida C1F8	Gainesville, FL	I
	T. sang 5 cl1†	Bulloch County, GA	I
Triatoma gerstackeri	Triatoma 2	Texas	I/IIa
	Triatoma 3	Texas	I
	TxTg2	Texas	I
Ring-tailed lemur	Nilda	St. Catherine's Island, GA	IIa
	Clarence	St. Catherine's Island, GA	IIa
	Meg	St. Catherine's Island, GA	IIa
Rhesus macaque	Monk RH89–40	Atlanta, GA (CDC)	I/IIa
	Texas Theis†	Not known	I
Nine-banded armadillo	Armadillo 1973†	New Orleans, LA	I
	GA Arm 20	Ossabaw Island, GA	IIa
	USA Armadillo†	Southern Louisiana	I
Striped skunk	GA Sk 1	Ludiwici, GA	IIa

*GA, Georgia; TX, Texas; LA, Louisiana, CA, California; TN, Tennessee; OK, Oklahoma; FL, Florida; AL, Alabama; MD, Maryland; CDC, Centers for Disease Control and Prevention; SC, South Carolina.
†Characterized by using multilocus enzyme electrophoresis (MLEE) or random amplified polymorphic DNA (RAPD) analysis (*7*).
‡Characterized by using microsatellite, 24S α rRNA, and COII genetic analysis (*11*).
§Characterized by using RAPD and MLEE analysis (*12*).
¶Characterized by using an unspecified method (*13*).
#Characterized by RAPD and mini-exon amplification (*7*).
